# Postpartum haemorrhage in midwifery care in the Netherlands: validation of quality indicators for midwifery guidelines

**DOI:** 10.1186/s12884-014-0397-8

**Published:** 2014-12-07

**Authors:** Marrit Smit, Kar-Li L Chan, Johanna M Middeldorp, Jos van Roosmalen

**Affiliations:** Department of Obstetrics, Leiden University Medical Centre, K6-35, PO Box 9600, Leiden, 2300 RC the Netherlands; EMGO Institute for Health and Care Research Department of Medical Humanities, VU University Medical Centre, Amsterdam, the Netherlands

**Keywords:** Post-partum hemorrhage, Home birth, Primary care, Midwifery, Quality indicators

## Abstract

**Background:**

Postpartum haemorrhage (PPH) is still one of the major causes of severe maternal morbidity and mortality worldwide. Currently, no guideline for PPH occurring in primary midwifery care in the Netherlands is available. A set of 25 quality indicators for prevention and management of PPH in primary care has been developed by an expert panel consisting of midwives, obstetricians, ambulance personal and representatives of the Royal Dutch College of Midwives (KNOV) and the Dutch Society of Obstetrics and Gynecology (NVOG). This study aims to assess the performance of these quality indicators as an assessment tool for midwifery care and suitability for incorporation in a professional midwifery guideline.

**Methods:**

From April 2008 to April 2010, midwives reported cases of PPH. Cases were assessed using the 25 earlier developed quality indicators. Quality criteria on applicability, feasibility, adherence to the indicator, and the indicator’s potential to monitor improvement were assessed.

**Results:**

98 cases of PPH were reported during the study period, of which 94 were analysed. Eleven indicators were found to be applicable and feasible. Five of these indicators showed improvement potential: routine administration of uterotonics, quantifying blood loss by weighing, timely referral to secondary care in homebirth and treatment of PPH using catherisation, uterine massage and oxytocin and the use of oxygen.

**Conclusions:**

Eleven out of 25 indicators were found to be suitable as an assessment tool for midwifery care of PPH and are therefore suitable for incorporation in a professional midwifery guideline. Larger studies are necessary to confirm these results.

## Background

Postpartum haemorrhage (PPH) is still one of the major causes of severe maternal morbidity and mortality worldwide. The rate of PPH has increased in recent years in many high income countries, including the United States, Canada, Australia, Norway, and Ireland [1-7]. In particular, PPH due to uterine atony has contributed to this rise, although the reasons for this remain unclear [3,7-10].

In the Netherlands, the overall incidence of PPH, defined as blood loss >1000 mL within 24 hours after birth, is 6% and this number is rising [11,12]. The definition of 1000 mL is often used in high-resource countries (such as the Netherlands) because a woman in good health can tolerate up to one liter of blood loss without showing early signs of shock.

Almost one third of Dutch women (32.7%) give birth in ‘primary care’ which is low risk care supervised by a midwife (99% of births) or general practitioner (1% of births). Of all births in primary care, 64% occur at home [12]. Of all women who give birth in primary midwifery care, the incidence of PPH is 3.4% [13]. There are various guidelines concerning prevention and management of PPH [14-16]. However, no guideline for PPH occurring in primary midwifery care in the Netherlands is available. In a primary care setting, limited hands-on assistance and the necessity of arranging ambulance transfer (in case of home birth) make the availability of a specific guideline for midwifery care essential. A set of 25 quality indicators for prevention and management of PPH in primary care has been developed by an expert panel consisting of midwives, obstetricians, ambulance personal and representatives of the Royal Dutch College of Midwives (KNOV) and the Dutch Society of Obstetrics and Gynecology (NVOG) [7]. This paper describes the performance of those quality indicators in clinical practice as an assessment tool for midwifery care and suitability for incorporation in a professional midwifery guideline. Validation is necessary to demonstrate the value of the set of indicators as an instrument for monitoring and improving prevention and management of PPH in primary care [17,18].

## Methods

Ethical clearance was granted by the Leiden University Medical Ethics Committee (P11.105).

### Data collection

From April 2008 to April 2010, 337 Dutch midwives who participated in the CAVE training (Pre-hospital Obstetric Emergency Course) were requested to participate in this study. The CAVE course is a post-graduate course which focuses on the identification and management of obstetric emergencies, including timely and adequate referral to hospital [19].The midwives who participated in the study originated from both rural and urban areas in the Netherlands. The midwives reported obstetric emergencies occurred in their practice such as PPH, shoulderdystocia, neonatal resuscitation, unexpected breech birth and umbilical cord prolapse. During twelve consecutive months, midwives received a monthly e-mail, linked to a password protected internet site. When obstetric emergencies were reported, participants were asked to fill out a detailed case registration form containing information on received care during pregnancy and birth and neonatal outcome. In addition, anonymous medical files, discharge letters and laboratory results were requested. Also, if ambulance transfer was necessary, details of transfer were requested from the ambulance services. The researchers contacted midwives, hospitals and ambulance services in order to obtain missing data. For this study, reported cases of PPH were collected and used for validation of 25 earlier developed quality indicators [7].

### Assessment of quality indicators

Each indicator was individually validated using the obtained case registration forms and assessed with respect to the following quality criteria: applicability, feasibility, adherence to the indicator and improvement potential [18,20,21].

*Applicability* was found if the indicator was applicable to a substantial amount of cases (>10 cases) [22]. Other quality criteria could not be assessed if an indicator was found not applicable and thus subsequently discarded [18]. *Feasibility* was considered to be present if the availability of administrative data required to assess the indicator could be abstracted from the data in >70% of cases. In contrast to other studies dictating a threshold of 75%, it was decided to lower the limit to 70%, as a PPH guideline is currently absent [21]. *Adherence to the indicator* was defined if data to fill the numerator and denominator of the indicator can be made available through data collection [18,20]. When an indicator is aimed to demonstrate changes in quality of care, there must be room for improvement [18]. *Improvement potential* was defined if less than 90% of the case registration forms met the requirements of the indicator [18,21].

Assessment of quality indicators was mostly unambiguous. For example, routine administration of uterotonics, use of oxygen and intravenous access were stated in every case registration form. However, ‘timely referral when blood loss is not ceasing’ contains potential subjectivity, and two assessors (KC, MS) therefore independently assessed cases. If there was no agreement, the case was discussed until consensus was reached.

### Statistical analysis

All cases of PPH were analyzed using IBM SPSS Statistics version 20 for Windows using Descriptive Statistics (Frequencies, Descriptives).

## Results

### Study population

During the study period, 98 cases of PPH in primary care were reported. Despite meticulous attempts to complete the files, four cases (4%) had to be excluded due to incomplete data, leaving 94 cases for analysis. Characteristics of the women with PPH are shown in Tables 1 and 2. The majority of women 72/94 (77%) gave birth at home and 22/94 (23%) gave birth in hospital or birthing clinic, all under supervision of the primary care midwife. Uterine atony was the primary cause of PPH in 64/94 women (68%). A retained or incomplete placenta was found in 27/94 women (29%) as primary cause of PPH. In three women (3%) vaginal or cervical injury was the primary cause of PPH.

**Table 1:** **Characteristics of 94 women with PPH in primary midwifery care**

***Characteristics***	***No. (n =94)***
Mean age, years (range)	31 (20–41)
Median gestational age, weeks (range)	40 (37 – 42)
Nulliparous (%)	44 (47)
Multiparous (%)	50 (53)
Home delivery (%)	72 (77)
Hospital delivery (%)	22 (23)
Median birth weight, gram (range)	3650 (2685–4620)
Median total blood loss, mL (range)	1800 (1000–7000)
Cause of PPH (%)	
- Retained placenta	44 (47)
- Uterine Atony	48 (51)
- Genital tract trauma	2 (2)
Median lowest haemoglobin, mmol/L, (range)	5.3 (3.3 - 8.6)
Median number of packed cells, units, (range)	0 (0–8)

**Table 2:** **Quality criteria for validation of 25 earlier developed quality indicators of PPH in primary midwifery care**

	***Category, indicators***	**Applicability**	**Feasibility**	**Amount of cases in adherence to indicator (%)**	**Improvement potential Yes, No or NA (not applicable) If adherence to indicator is <90%**
		**n**_**patients**_	**% of patients with missing values**		
		**If number of patients is >10**	**If availability of data is >70%**		
	*Prevention*				
	**Antenatally: identify**	94	0		No
1.	**elevated- or high risk and agree on preventive strategies.**				
	- No elevated- or high risk of PPH identified			85 (90)	
	- Elevated- or high risk of PPH identified			9 (10)	
	○ Referred to secondary care			9 (100)	
	○ Not referred to secondary care			0 (0)	
	high risk and agree (or adjust) on preventive strategies.				
2.	At birth: identify elevated- or high risk	94	100	NA	NA
3.	If high risk is assessed: have birth occur in hospital supervised by the obstetrician.	94	100	NA	NA
4.*	**Routinely administer uterotonics (at least 5 IU oxytocin intramuscular).**	94	0		Yes
	- Yes, at least 5 IU oxytocin			54 (57)	
	- No			40 (43)	
	*In case of blood loss >500 mL, without signs of shock the midwife should;*				
5. **	**Objectify blood loss by weighing.**	94	28		Yes
	- Yes			68 (72)	
	- No/unknown			26 (28)	
6. ***	**Homebirth: in case of retained placenta; refer to secondary care after 30 minutes.**	35	0		Yes
	- Referral <35 minutes			13 (37)	
	- Referral >35 minutes			22 (63)	
7. ***	Midwifery supervised hospital birth: in case of retained placenta; refer to secondary care after 30 minutes.	9/ No	11		NA
	- Referral <35 minutes			3 (33)	
	- Referral >35 minutes			5 (56)	
8.	**Home birth; if blood loss is not ceasing, refer to secondary care.**	35	0		No
	- Timely referral			32 (91)	
	- No timely referral			3 (9)	
9.	**Midwifery supervised hospital birth if blood loss is not ceasing, refer to secondary care.**	13	0		No
	- Timely referral			13 (100)	
	- No timely referral			0 (0)	
10.	**Treat PPH as uterine atony until proven otherwise.**	94	0		Yes
	A Catheter			77 (82)	
	B Uterine massage			66 (70)	
	C Oxytocin			74 (79)	
	D Combination of catheter, uterine massage and oxytocin			53 (56)	
11.	**Post placental: if blood loss is not ceasing despite administration of uterotonics; examine for vaginal and perineal lesions**	94	1	93 (99)	No
	*In case of PPH of >1000 mL and/or signs of shock, the midwife should;*				
12.	**Inform the secondary caregiver (obstetrician).**	94	0		No
	- Yes			92 (98)	
	- No			2 (2)	
13.	**Start an intravenous line and supply with fluids, using 0,9% sodium chloride**	94	1		No
	A. Midwife			22 (23)	
	B. Ambulance personnel			47 (50)	
	C. Hospital personnel (gynecologist or nurse)			21 (22)	
	D. No intravenous line given			3 (3)	
	E. Total given			91 (97)	
14	Monitor vital signs frequently.	94	60		NA
^β^	A Blood pressure			14 (15)	
	B Pulse			1 (1)	
	C Blood pressure &			23 (25)	
	D pulse				
	E Total reported			38 (40)	
15.	**Regardless of oxygen saturation, provide patient with 10–15 liter oxygen via non-rebreathing mask.**	94	0		Yes
	- Yes			10 (11)	
	- No			84 (89)	
	*In case of PPH of >1000 mL with signs of shock and/or >2000 mL blood loss the midwife should;*				
16.	In case of persisting hemorrhaging with signs of shock, perform uterine and/ or aortal compression.		94	100/No	NA
17.	Secure a second intravenous line (14 gauge).	3/ No	67		NA
	- Yes			0 (0)	
	- No			1 (33)	
18.	If the patient has reduced consciousness due to hypovolemic shock, call for (paramedic) assistance in order to establish an open airway.	3/ No	100	NA	NA
19.	Immediately transfer patient to secondary care.	3/ No	0		NA
	- Yes			2 (67)	
	- No			1 (33)	
	*Concerning cooperation, training and documentation*				
20.	Within every regional obstetric collaboration† a regional PPH protocol should be present, based on the national guidelines.	94	100	NA	NA
21.	A regional PPH protocol should be the basis of regular audits	94	100	NA	NA
22.	Every midwife should be aware that ambulance transportation in case of PPH or retained placenta is always of the highest urgency category (A1).	94	32		NA
	- A1 (arrival at patient			51 (54)	
	- within 15 minutes)				
	- A2 (arrival at patient within 30 minutes)			13 (14)	
23.	After each PPH with >2000 mL blood loss, the multidisciplinary team should debrief the situation.	3/ No	100	NA	NA
24.	Within the regional obstetric collaboration† an annual training in obstetric emergencies should be provided.	94	100	NA	NA
25.	In a homebirth situation, anticipation on possible ambulance transport is necessary; make sure the patient is at an accessible place for (all) caregivers in time.	94	100	NA	NA

*Within 3 minutes after birth, at least 5 IU (international units) oxytocin intramuscular is given.

**Estimated or measured blood loss before referring to secondary care.

***In case of retained placenta, the midwife called the obstetrician within 35 minutes after birth to refer and, in case of home birth, ambulance assistance is requested and on the way.

^β^A single documentation of pulse and blood pressure would meet the requirements of this indicator.

† Regional obstetric collaboration; a quarterly meeting with obstetricians and midwifery practices within a region in the Netherlands where policy, collaboration and practical agreements are discussed.

NA, not applicable (Applicable and/or feasible indicators are in bold).

Five indicators were only found relevant in <10 cases and therefore inapplicable. Nine indicators were found not feasible; the administrative data required to evaluate the indicator were available in less than 70% of cases.

Adherence to the indicator was analyzed for the remaining 11 indicators. Five of these indicators showed to have improvement potential, with an adherence to the indicator less than 90%, and therefore indicating room for improvement. Assessment of ‘timely referral’ led to discussion in two cases, however, consensus was reached after discussion.

## Discussion

Aim of this study was to assess the performance of the 25 quality indicators of PPH in primary midwifery care. After applying the indicators to each of the 94 cases, 11 indicators could be validated to measure care provided by midwives to prevent and manage PPH in primary care. Five of these (5/11) showed potential to be used to monitor improvement of the quality of care in our study.

PPH guideline development and implementation is an important (worldwide) topic as the incidence is still rising [23]. The present guidelines vary greatly per country, as evidence and background on which the guidelines are drawn upon differs (for example, the presence of primary midwifery care). And practical matters such as geographic landscape (e.g. road network) and proximity to hospital are of influence on the approach of PPH. This study forms an important step in the development of a guideline for prevention and management of PPH in primary midwifery care. An important strength of this study is the use of effective methods such as a RAND modified Delphi procedure and applying validated quality criteria (applicability, feasibility, adherence to the indicator and improvement potential) [18,20,21]. As we thoroughly followed these steps, these indicators are valid, usable in clinical practice and form an important basis in guideline development.

Blood loss over 2000 mL at time of referral is a rare phenomenon, especially in primary midwifery care and occurred in only three of our 94 cases. Further exploration of the indicators related to blood loss over 2000 mL is recommended with more cases of such high blood loss. Nine indicators were found not feasible. Information about the indicators was either partially or completely missing in case registration forms or medical files, suggesting documentation of midwives may need improvement. Further research is needed to explore whether specific care was not noted or care was indeed provided but not documented in the medical file. The remainder of 14 indicators (those who were found not feasible and/or applicable) were selected through a meticulous RAND modified Delphi procedure and therefore have potential to be incorporated in a guideline. They may not be suitable as tools for quality improvement in its present form. A larger study, however, may show improvement potential for these indicators. Although these items are not suitable as a quality tool in the present form, they should not be discarded in incorporating in a guideline, as they are validated [7]. Our small sample is a limitation of the study. A possible selection bias is another limitation. Only midwives who successfully finished the CAVE course reported cases. One can assume that these participants perform very well in case of PPH as they were recently trained. Further research should also include midwives, who did not participate in the CAVE training.

## Conclusions

This is the first study describing quality indicators particularly for PPH in primary midwifery care in the Netherlands. Eleven out of 25 indicators were found to be suitable as an assessment tool for midwifery care of PPH and are therefore suitable for incorporation in a professional midwifery guideline.
